# Photoluminescence decay rate of silicon nanoparticles modified with gold nanoislands

**DOI:** 10.1186/1556-276X-9-165

**Published:** 2014-04-04

**Authors:** Viktor Dan’ko, Katerina Michailovska, Ivan Indutnyi, Petro Shepeliavyi

**Affiliations:** 1 V. Lashkaryov Institute of Semiconductor Physics National Academy of Science of Ukraine, 45 Prospect Nauky, Kyiv 03028, Ukraine

**Keywords:** Si nanoparticle, Nanostructure, Photoluminescence, Localized surface plasmon, Plasmon-induced enhancement, Nanoisland film, 78. 67. Bf, 78.55.-m

## Abstract

We investigated plasmon-assisted enhancement of emission from silicon nanoparticles (ncs-Si) embedded into porous SiO_*x*_ matrix in the 500- to 820-nm wavelength range. In the presence in the near-surface region of gold nanoisland film, ncs-Si exhibited up to twofold luminescence enhancement at emission frequencies that correspond to the plasmon resonance frequency of Au nanoparticles. Enhancement of the photoluminescence (PL) intensity was attributed to coupling with the localized surface plasmons (LSPs) excited in Au nanoparticles and to increase in the radiative decay rate of ncs-Si*.* It has been shown that spontaneous emission decay rate of ncs-Si modified by thin Au film over the wide emission spectral range was accelerated. The emission decay rate distribution was determined by fitting the experimental decay curves to the stretched exponential model. The observed increase of the PL decay rate distribution width for the Au-coated nc-Si-SiO_*x*_ sample in comparison with the uncoated one was explained by fluctuations in the surface-plasmon excitation rate*.*

**PACS:**

78. 67. Bf; 78.55.-m

## Background

Enhancement of the intensity and emission rate of quantum emitters is of significant interest during the past decade. One of the approaches to enhance luminescence efficiency of low-dimensional materials is to realize the coupling of electronic excitation in quantum dots and wells with the surface plasmons (SPs) supported by metal nanostructures. Metal nanostructures can be of two types: planar metal films and non-planar metal nanostructures such as nanoparticle arrays and thin semicontinuous metal films consisting of disorder-shaped nanostructures. When a planar metal film is placed above a luminescent material, the emission decay rate of it increases due to excitation of the propagating mode surface plasmons [1,2]. Surface plasmon excitations in bounded geometries such as nanostructured metal particles are localized surface plasmons (LSPs). The resonant excitation of LSPs on the surface of nanostructured metallic particles by an incident light causes strong light scattering and absorption and enhanced local electromagnetic fields [3]. In non-planar metal nanostructures, localized modes of the SPs play an important role in changing the decay rate of luminescent material. The decay rate characteristics for non-planar metal nanostructures are different from those for planar films, e.g., strong dependence of the decay rate on wavelength [4], polarization [5], and fluctuation of decay rate distribution [6]. Changes in the photoluminescence (PL) intensity and the spontaneous decay rate due to deposition of metal nanostructures are observed in a semiconductor nanocrystals and organic materials [7-9]. It has been shown that the PL intensity of silicon nanocrystals can be considerably enhanced by placing an Ag island array with different sizes and pitches [10]. Further, polarization-selective enhancement of PL was realized by using an anisotropic metal structure [11]. There are no investigations on the effect of metal nanoparticles on the radiative recombination of silicon nanoparticles in anisotropic dielectric matrix. In this paper, we studied the emission decay rate of ncs-Si embedded into the SiO_*x*_ matrix possessing a porous column-like structure covered with a thin Au film. We show evidence of enhanced radiative recombination in Au-coated nc-Si-SiO_*x*_ structures resulting from the coupling between ncs-Si and LSPs excited in Au nanoparticles.

## Methods

### Experimental

The investigated samples were produced by thermal evaporation of Cerac Inc., Milwaukee, WI, USA, silicon monooxide SiО with 99.9% purity in vacuum (the residual pressure (1…2)∙10^−3^ Pa). During glance angle-SiО deposition, the substrate (polished Si wafer) was oriented at the angle *α* = 75° between the normal to the substrate surface and the direction to the evaporator. The thickness of oblique deposited films was chosen with the range 400…600 nm. Because of additional oxidation by residual gases during evaporation of SiO, the compositionally non-stoichiometric SiO_*x*_ (*x* ~ 1.5) films were deposited in the vacuum chamber. After their deposition, the porous SiO_*x*_ films were annealed in the vacuum chamber at 975°C for 15 min to grow ncs-Si.

**Figure 1:**
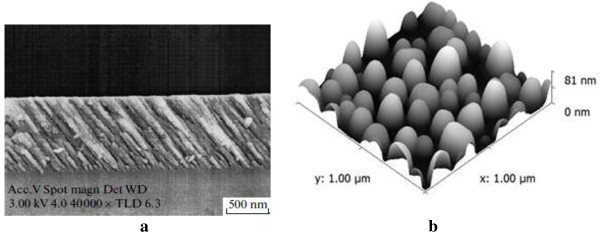
**Cross-section view and AFM topology. (a)** SEM micrograph of SiO_*x*_ film cross-section and **(b)** AFM topology of the surface of 5 nm gold film annealed at 450°C.

The structure of obliquely deposited SiO_*x*_ films was studied by SEM apparatus (ZEISS EVO 50XVP, Oberkochen, Germany). In Figure 1a, the cross-sectional view of SiO_*x*_ film oblique deposited on silicon wafer is shown. As can be seen in the figure, the investigated SiO_*x*_ films have a porous inclined pillar-like structure with the pillar diameters of 10 to 100 nm. The porosity of films depends on the angle of deposition and equals to 53% for *α* = 75°. High-temperature annealing of these films does not change the porosity and pillar-like structure of the samples [12].

The obtained nc-Si-SiO_*x*_ structures were passivated in the HF vapor, which results in the enhancement of the PL intensity by approximately 200 times [13]. Thin Au layers were deposited on one part of the passivated nc-Si-SiO_*x*_ structures by thermal evaporation and then annealed at 450°C in vacuum. The mass thickness of the Au layers was about 5 nm. Studying topology of the Au layers was carried out with an atomic force microscope (AFM) NanoScope IIIa (produced by Digital Instrument, Tonawanda, NY, USA). An axonometric AFM image of the Au layer surface is presented in Figure 1b. One can see that the Au layer is semicontinuous and consists of nanoislands.

The photoluminescence spectra were recorded at room temperature using a system based on a ZMR-2 monochromator equipped by a photomultiplier tube and detection system. The PL spectra were normalized to the spectral sensitivity of the experimental system. The PL signal was excited by radiation of a N_2_ laser at the wavelength 337 nm. The excitation and detection of PL emission was carried out through the front side of samples. In PL spectra, we took into account the transmittance of exciting light and PL emission through an Au film. The absorbance properties of reference Au films, evaporated on glass substrates, were investigated by means of transmission and reflection measurements by using the spectrometer NanoPlasmon-2048 VIS, Boston, MA, USA.

Decay curve measurements were performed using the N_2_ laser with the pulse duration 9 ns and pulsed oscillograph C1-54. The system time resolution was 0.5 μs.

## Results and discussion

**Figure 2:**
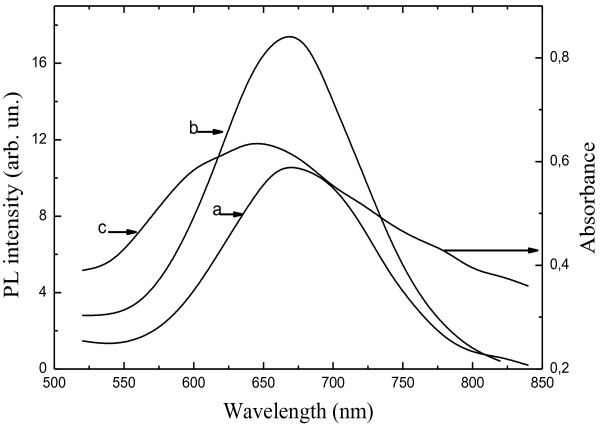
**PL spectra of nc-Si-SiO**_***x***_**structures. (a)** Without Au layer, **(b)** with Au 5 nm layer, and **(c)** absorbance spectra for Au 5 nm film, annealed at 450°C.

To understand the effect of Au nanoparticles on the PL emission of ncs-Si embedded into SiO_*x*_ matrix, we measured the PL spectra of nc-Si-SiO_*x*_ structures with and without thin Au layer. Figure 2 shows the PL spectrum of the nc-Si-SiO_*x*_ structures uncoated (a) and coated (b) by Au film. The uncoated nc-Si-SiO_*x*_ structure exhibits strong PL emission within the wavelength range 500 to 820 nm with a peak near 660 nm, which could be attributed to exciton recombination in ncs-Si [14]. A more than twofold increase of the PL intensity from the structure covered with Au layer was clearly observed. A maximum PL enhancement factor of 2.2 was observed at 640…660 nm (after taking into account the transmittance of exciting light and PL emission through the Au film).

Figure 2c shows absorbance spectra of Au layer evaporated on glass substrate simultaneously with that evaporated on the nc-Si-SiO_*x*_ structure. The absorbance spectra of Au film presented the typical wide absorption band in the visible region of the spectrum. Maximum of this band at 640…660 nm corresponds to the resonance of the LSPs excited in Au nanoparticles [15]. Close peak positions of the ncs-Si emission and absorption of Au nanoparticles indicate that excitons generated in ncs-Si could effectively couple to electron vibrations at the surface of Au nanoparticles because the emission frequency is matched to the plasmon resonance one. The PL enhancement can arise from the increased external quantum efficiency of ncs-Si PL (correlates to an increase of the radiative decay rate). When exciton dipole moment of nc-Si strongly couple to the local electric field of LSPs in Au layer, the nc-Si-LSP coupling, according to Fermi's golden rule, increases the radiative recombination rate [16,17], resulting in increase of radiative efficiency. A more direct demonstration of enhanced exciton recombination involved comparative measurements of the PL decay rate from investigated structures.

**Figure 3:**
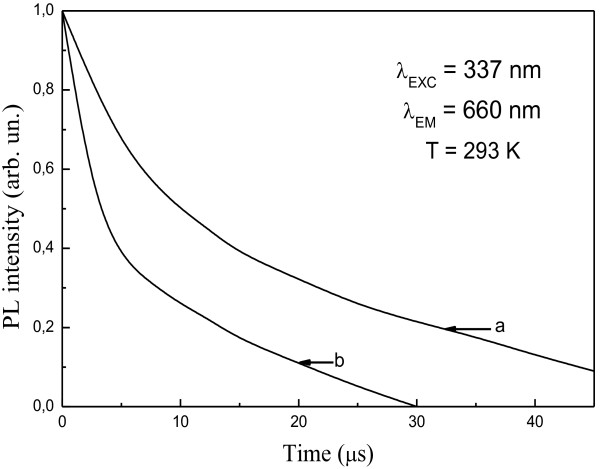
**PL decay curves measured at *λ*** **= 660 nm. (a)** nc-Si-SiO_*x*_ structure not covered with Au layer; **(b)** nc-Si-SiO_*x*_ structure covered with Au 5 nm layer.

Time-resolved PL measurements were performed using the same luminescent uncoated and Au-coated nc-Si-SiO_*x*_ samples. Figure 3 shows the ncs-Si PL decay curve measured for the uncoated (a) and Au-coated (b) nc-Si-SiO_*x*_ samples at 660 nm. One can see that the PL decay of the Au-coated samples is accelerated as compared to that in the uncoated ones. All experimental curves of PL decay might be described well by a stretched exponential function:

(1) $$I_{\mathrm{PL}}\left(t\right)=Ct^{\beta -1}\exp\left[-{\left(\frac{t}{\tau_{0}}\right)}^{\beta }\right]$$

where C, *τ*_0_, and *β* are a constant, decay time, and stretched parameter *(*0 < *β* ≤ 1*)*, respectively. It is known that this model is widely used for the decay curve analysis of Si nanocrystals [18]*.* The least squares fit of Equation 1 to experimental data brings values of *τ*_0_ and *β.* The obtained decay times *τ*_0_ were equal to 16 and 5.2 μs for uncoated and Au-coated nc-Si-SiO_*x*_ samples, respectively. It was determined also that the dispersion parameter *β* for nc-Si-SiO_*x*_ structures without and with the gold layer decreased from 0.76 to 0.53, respectively. The latter *β* value corresponds to a larger distribution width of decay rates for Au-nc-Si-SiO_*x*_ interface. In the case of stretched exponential relaxation function, the PL decay might be analyzed more thoroughly by recovering the distribution of recombination rates [18]. So, having the constants of *τ*_0_ and *β*, taken from experimental data fit to (1), it is possible to obtain the average decay time constant *< τ>*, which can be defined by:

(2) $$\langle \tau \rangle =\tau_{0}\beta^{-1}Ã\left(\beta^{-1}\right)$$

where *Г* is the gamma function. The average decay times < *τ* > were equal to 18.9 μs for the uncoated and 9.4 μs for Au-coated samples. It is seen that the parameter *β* and decay time decrease for nc-Si-SiO_*x*_ structures coated with Au layer. Accordingly, the decay rate (*k = τ*_0_^−1^) at 660 nm is increased from 6.25 × 10^4^ s^−1^ for uncoated to 19.2 × 10^4^ s^−1^ for the Au-coated samples, an enhancement by a factor approximately 3.

**Figure 4:**
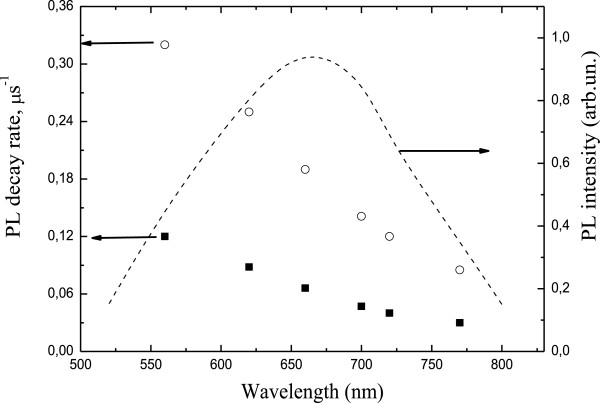
**Wavelength dependence of the PL decay rates of nc-Si-SiO**_***x***_**structure.** Without Au layer (solid squares) and with Au layer (open circles). Dashed curve is PL spectra of nc-Si-SiO_*x*_ structure.

In order to investigate the wavelength dependence of the decay rates, we measured PL decay curves in a whole emission wavelength range. These results are shown in Figure 4. The decay rate increases as the emission wavelength is shortened both for uncoated (a) and the Au-coated (b) nc-Si-SiO_*x*_ samples due to the quantum size effect.

**Figure 5:**
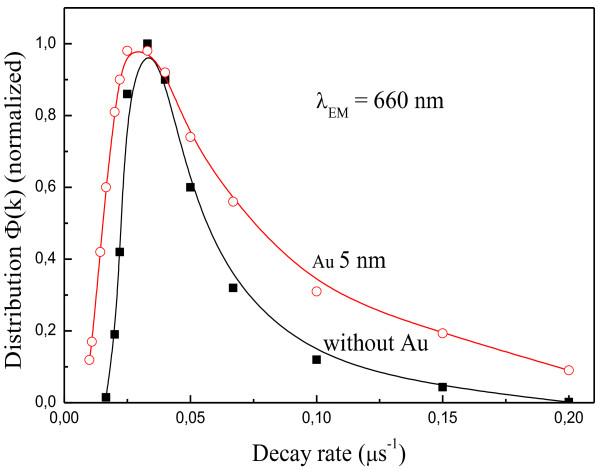
**Decay rate distributions of nc-Si-SiO**_***x***_**structures with and without Au 5 nm layer.**

Using the values of *τ*_0_ and *β* measured at *λ* = 660 nm, we calculated the asymptotic form of the decay rates probability density function *Ф*(*k*) that may be obtained by the saddle point method [19]:

(3) $$Ф\left(k\right)=\frac{\mathrm{a\tau}}{\sqrt{2\pi \beta }}*{\left(\mathrm{k\tau}\right)}^{-1-a/2}*\exp\left[-\left(\mathrm{k\tau}\right)-a\right]$$

where *a* = *β*(1 − *β*)^−1^ and *τ* = *τ*_0_[*β*(1 − *β*)^1/*a*^]^−1^. Figure 5 shows the *Ф*(*k*) distributions calculated from Equation 3 for nc-Si-SiO_*x*_ and Au-nc-Si-SiO_*x*_ samples. We can see increase in the decay rate distribution width for the Au-coated nc-Si-SiO_*x*_ sample in comparison with the uncoated one. A possible reason of the *Ф*(*k*) broadening may be the uncertainty in the distance between deposited Au nanoparticles and nc-Si embedded into porous SiO_*x*_ matrix because the surface of the HF vapor-etched nc-Si-SiO_*x*_ layer has a significant roughness. Such an uncertainty in the metal-emitter distance could lead to fluctuations in the local density of optical states (LDOS). This is because the change in the LDOS, due to the surface plasmon excitation, is strongly dependent on this distance [20], i.e., the excitation rate of LSPs could be largely influenced by the uncertainty in the metal-emitter distance. Therefore, the larger decay rate fluctuation is attributed to the fluctuations in the surface-plasmon excitation rate.

Other model used for the statistical analysis of the time-resolved emission from the assembly of semiconductor quantum dots was proposed by van Driel et al. [21], which takes into consideration the log-normal distribution of decay rates. This model was used under studies of spontaneous emission decay rate, an assembly of Si nanocrystals in porous silicon (PSi) near semicontinuous gold films [22]. For the Au/PSi samples, the log-normal model gave a good fit with the experimental dates. It has been shown that PL decay rates also strongly modified upon deposition of a thin Au film. The decay rate fluctuation in Au/PSi samples was related to the fluctuations in the LDOS.

## Conclusions

We investigated the photoluminescence spectra of the silicon nanoparticles, embedded into porous SiO_*x*_ matrix, coated by Au-nanoisland layer. It has been shown that the spontaneous emission decay rate of the excited ncs-Si in the sample coated by Au nanoislands was accelerated. Close peak positions of the nc-Si emission and absorption of Au nanoparticles indicate that excitons generated in ncs-Si could effectively couple to the local surface plasmons excited at the surface of Au nanoparticles and increase the radiative recombination rate. We studied also the wavelength dependence of the PL decay rates in the samples with and without Au layer. The emission decay rate distribution was determined by fitting of the experimental decay curves within frameworks of the stretched exponential model. It was supposed that for the Au-coated nc-Si-SiO_*x*_ samples, the larger width in the decay rate distribution might be attributed to the fluctuations in the surface-plasmon excitation rate due to the uncertainty in the metal-emitter distance.

## Abbreviations

AFM: atomic force microscope; LDOS: local density of optical states; LSPs: localized surface plasmons; PL: photoluminescence; SEM: scanning electron microscopy; SPs: surface plasmons.

## Competing interests

The authors declare that they have no competing interests.

## Authors’ contributions

The idea of the study was conceived by VD and II. PS and II produced investigated structure. KM performed the photoluminescence measurements as well as calculation and initiated the first draft of the manuscript. All authors read and approved the final manuscript.
